# Long-term efficacy and safety of different biologics in treatment of chronic rhinosinusitis with nasal polyps: A network meta-analysis

**DOI:** 10.1016/j.bjorl.2025.101633

**Published:** 2025-04-25

**Authors:** Han Chen, Lin Wang, Jisheng Zhang, Xudong Yan, Longgang Yu, Yan Jiang

**Affiliations:** The Affiliated Hospital of Qingdao University, Department of Otolaryngology Head and Neck Surgery, Qingdao, China

**Keywords:** Biologics, Chronic rhinosinusitis with nasal polyps, Long-term efficacy, Network meta-analysis

## Abstract

**Objectives:**

Direct comparison of the long-term effectiveness and safety of biologics for Chronic Rhinosinusitis with Nasal Polyps (CRSwNP) is lacking. The study aimed to compare the long-term efficacy and safety of various biologics in the management of CRSwNP.

**Methods:**

The PubMed, Embase, Cochrane Library, and Web of Science databases were searched from database inception to March 2024, to identify all relevant literature on the use of biologics for CRSwNP. The research protocol was registered on PROSPERO.Two independent reviewers screened the studies, extracted the data, and performed a quality assessment using the Cochrane Risk of Bias tool. Network meta-analysis was conducted using STATA 17.0 and Review Manager (Version 5.3).

**Results:**

Six studies with a minimum follow-up period of 52-weeks were analyzed, demonstrating the superior long-term efficacy of dupilumab compared to the other three biologics. The surface under the cumulative ranking curve were 100% for Nasal Polyp Score (NPS), 72.7% for Sino-Nasal Outcome Test-22 (SNOT-22), 94.6% for Visual Analogue Scale (VAS), and 100% for Nasal Congestion Score (NCS). In the comparison of NPS, dupilumab showed improvements of 1.84 (95% Confidence Interval [95% CI 0.78, 2.91]) over mepolizumab and 2.31 (95% CI 0.99, 3.63) over benralizumab. Among the symptom scores evaluated, only dupilumab achieved a significant improvement in NCS compared to benralizumab, with an improvement of 0.74 (95% CI 0.86, 1.19). No significant differences in adverse events was observed between biologics treatment or versus placebo.

**Conclusion:**

Treatment with dupilumab in patients with CRSwNP has shown superior long-term efficacy in reducing NPS and various symptom scores compared to other biologics. However, the results should be interpreted with caution due to the heterogeneity of patient characteristics across the included studies (e.g., disease severity, history of surgery, use of oral corticosteroids).

## Highlights

• Dupilumab tops for CRSwNP in network meta-analysis, reducing NPS and symptoms.
• Biologics exhibit comparable long-term safety to placebo.
• Personalized biologic choice crucial in CRSwNP; prolonged studies needed.

## Introduction

Chronic Rhinosinusitis with Nasal Polyps (CRSwNP) is a burdensome chronic inflammatory disease, affecting 0.5%–4.3% of the global population, characterized by bilateral nasal polyps and sinusitis.^1^ Current treatment guidelines for CRSwNP recommend approaches such as nasal corticosteroids and nasal irrigation; however, a significant proportion of patients, approximately one-third, exhibit a suboptimal response to these standard treatments, necessitating more aggressive interventions like oral corticosteroids and sinus surgery.^2^ Despite these interventions, the recurrence rate remains high, with a 40% recurrence after 18 months following successful surgery,^3^ and prolonged use of oral corticosteroids is associated with serious side effects.

The pathophysiology of CRSwNP is complex, with type 2 inflammation and eosinophilic inflammation being identified as key risk factors for disease recurrence, associated with elevated levels of Interleukin (IL)-4, IL-5, IL-13, and IgE.^4^ This has led to an urgent need for novel treatment modalities that can effectively target these inflammatory pathways.

In recent years, biological agents, particularly monoclonal antibodies, have emerged as promising therapeutic options for CRSwNP. These agents target the effector products of type 2 inflammation, such as IgE, IL-4, IL-5, IL-13, and their receptors, and have demonstrated both efficacy and safety in numerous multicenter double-blind placebo-controlled clinical trials worldwide.^5^, ^6^, ^7^

Despite the growing body of evidence supporting the use of biological agents, there is a paucity of studies directly comparing the long-term efficacy and safety of these agents in the treatment of CRSwNP. Meier et al.^8^ reported success rates of 79% for mepolizumab, 50% for omalizumab, and 50% for benralizumab, but the study was limited by a small sample size and variable treatment durations. Gomez et al.^9^ conducted an initial multicenter study comparing dupilumab and omalizumab, yet the treatment duration was restricted to 24 weeks. Additionally, previous meta-analyses^10^, ^11^, ^12^ primarily focused on short-term efficacy at 24-weeks, and a network meta-analysis^13^ did not specify treatment duration when assessing the efficacy of different monoclonal antibodies and aspirin desensitization drugs.

The present network meta-analysis aims to address these gaps in the literature by investigating the long-term efficacy and safety of different biological agents in the treatment of CRSwNP. This comprehensive analysis will provide valuable insights for clinicians in selecting the most appropriate treatment strategies for their patients.

## Methods

### Search strategy

This study has been registered on the International Prospective Register of Systematic Reviews. A comprehensive search was performed in PubMed, Embase, Cochrane Library, and Web of Science databases to identify all literature on biological agents for treating CRSwNP from the inception of the databases to March 2024. The search utilized a combination of subject terms and free text terms, with subject terms including “Sinusitis”, “nasal polyps”, “biological therapy”, and “monoclonal antibodies”, and was limited to English language publications (Table S1).

### Inclusion and exclusion criteria for literature

Inclusion Criteria: (1) Population: Adult patients (≥18 years old) diagnosed with CRSwNP through Computed Tomography (CT) or endoscopy, who continue to experience sinusitis despite prior treatment with steroids or surgery; (2) Follow-up period: A minimum of 52 weeks of follow-up from the initiation of treatment (assessing the long-term efficacy of drug therapy for at least 1 year)^2^; (3) Interventions: All forms of biological agents utilized for treating CRSwNP; (4) Control group: Placebo. Exclusion Criteria: patients with fungal sinusitis, cystic fibrosis, or other conditions that could impact the outcomes.

### Data extraction and quality assessment

Two researchers independently conducted the screening of included studies, data extraction, and resolution of any discrepancies in data extraction or quality assessment through discussion. The data extraction encompassed: 1) Basic information of the included studies; 2) Demographic characteristics and intervention protocols of the study subjects; 3) The primary outcome measures, which include efficacy (Nasal polyp score, NPS) and safety (Adverse events, AEs, quantified by the number of subjects experiencing one or more adverse reactions), while the secondary outcome measures encompass the Sino-Nasal Outcome Test-22 (SNOT-22), Visual Analogue Scale (VAS), and Nasal Congestion Score (NCS). The detailed descriptions of the outcomes were provided in Table S2. Quality assessment was performed by the two researchers using Review Manager (Version 5.3) in accordance with the risk of bias assessment tool recommended by Cochrane.^14^

### Statistical analysis

A network meta-analysis was performed utilizing Stata 17.0 software and based on Bayesian hierarchical models. Continuous variables lacking dimensionless differences in the study were assessed using the weighted mean difference, with a significance level set at α = 0.05, and 95% Confidence Intervals (95% CI) were computed. Consistency and inconsistency model tests were conducted on the data to ascertain overall inconsistency. The Surface Under the Cumulative Ranking Curve (SUCRA) was employed to rank the efficacy of various biological agents.

## Results

### Search results and characteristics of the literature

**Fig. 1:**
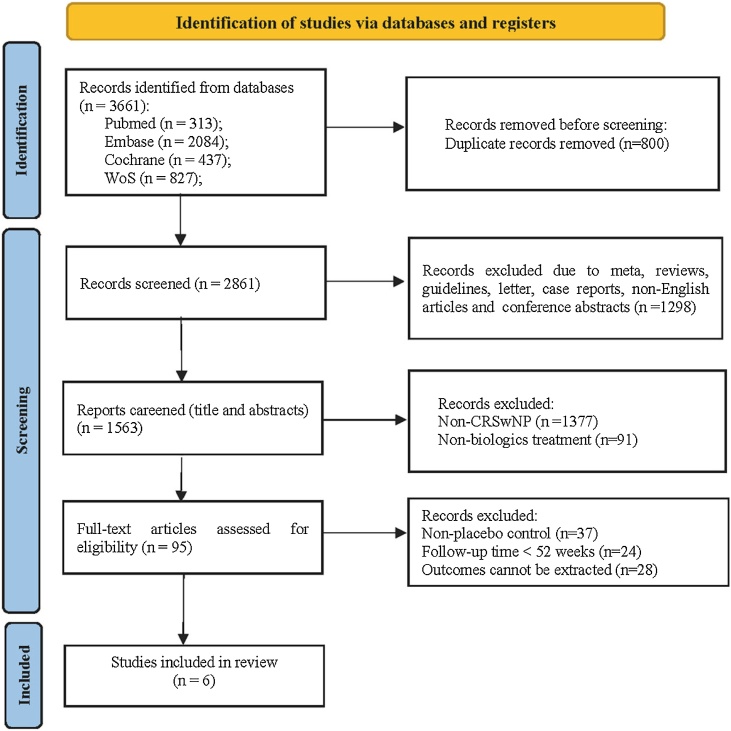
Flow diagram of study selection of the relevant articles.

A total of 3,661 articles were initially retrieved, and following a step-by-step screening process, six articles were ultimately included (Fig. 1). These comprised five Randomized Controlled Trials^15^, ^16^, ^17^, ^18^, ^19^ (RCTs) and one RCT subgroup^20^ (derived from the NAVIGATOR study). The randomization scheme of the NAVIGATOR study stratified based on the presence or absence of CRSwNP at baseline, aligning with the principles of randomization. Therefore, we included the subgroup data of patients with comorbid CRSwNP. The basic characteristics of the included literature are detailed in Table 1, encompassing a total of 1,566 adult patients involved in phase III clinical trials of CRSwNP and four types of biological agents, namely benralizumab (anti-IL-5Rα), dupilumab (anti-IL-4Rα), mepolizumab (anti-IL-5), and tezepelumab (anti-TSLP).

**Table 1:** Main characteristics of the studies included in the meta-analysis.

Study (Year)	Population	Comorbidity	Intervention	Placebo	Overall population		Age (Mean ± SD)		Male, n (%)		Follow-up length
					Biologist	Control	Biologist	Control	Biologist	Control	
Claus Bachert (2019) ^15^	CRSwNP	Asthma (59.6%) in Dupilumab group and 59% in the placebo group	(a) Dupilumab 300 mg subcutaneously every 2-weeks for 52-weeks; (b) dupilumab 300 mg subcutaneously every 2-weeks for 24-weeks, followed by every 4-weeks until 52-weeks	Injection, same dose and frequency	(a)150; (b)145	153	(a) 51.35 ± 14.2; (b) 52.65 ± 15.72	52.6 ± 12.7	(a) 97 (65%); (b) 87 (60%)	95 (62%)	52 weeks
Joseph K Han (2021) ^16^	CRSwNP (all had undergone endoscopic sinus surgery)	Asthma (67.9%) in Mepolizumab group and 74.1% in the placebo group	Mepolizumab 100 mg subcutaneously every 4-weeks for 52-weeks; Background therapy: intranasal MFNS at stable dose. Systemic corticosteroids used when needed.	Injection, same dose and frequency	206	201	48.6 ± 13.6	48.9 ± 12.5	139 (67%)	125 (62%)	52 weeks
Shigeharu Fujieda (2021)^18^	CRSwNP	Asthma (58.8%) in Dupilumab group and 68.8% in the placebo group	(a) Dupilumab 300 mg subcutaneously every 2-weeks for 52-weeks; (b) Dupilumab 300 mg subcutaneously every 2-weeks for 24-weeks, followed by every 4-weeks until 52 weeks	Injection, same dose and frequency	(a)16; (b)17	16	(a) 50.5 ± 10.5; (b) 54.1 ± 11.8	55.9 ± 10.4	(a) 12 (75%); (b) 8 (47.1%)	10 (62.5%)	52 weeks
Claus Bachert (2021) ^17^	CRSwNP	Asthma (68.6%) in Mepolizumab group and 67% in the Benralizumab group	Benralizumab 30 mg subcutaneously every 4-weeks for the first 3 doses and every 8-weeks thereafter till 48-weeks Background therapy: intranasal MFNS at stable dose.	Injection, same dose and frequency	207	203	50.1 ± 12.4	50.2 ± 13.9	142 (68.6%)	121 (59.6%)	56 weeks
Tanya M Laidlaw (2023) ^20^	CRSwNP	Asthma were both 100% in placebo group and Tezepelumab group	Tezepelumab 210 mg subcutaneously every 4-weeks for 52-weeks;	Injection, same dose and frequency	62	56	51.2 ± 13.3	50.4 ± 12.6	25 (40.3%)	28 (50%)	52 weeks
Martin Desrosiers (2023) ^19^	CRSwNP (all had undergone endoscopic sinus surgery)	Asthma (67.9%) in Mepolizumab group and 74.1% in the placebo group	Mepolizumab 100 mg subcutaneously every 4-weeks for 52-weeks; Background therapy: intranasal MFNS at stable dose. Systemic corticosteroids used when needed.	Injection, same dose and frequency	69	65	48.6 ± 13.6	48.9 ± 12.5	46 (67%)	40 (62%)	52 weeks

### Quality assessment and publication bias test

**Fig. 2:**
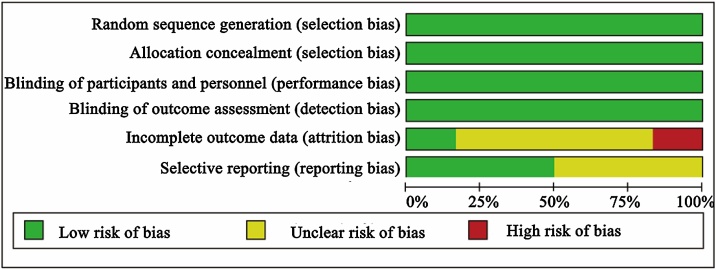
Quality assessment graph of risk of bias.

A bias risk graph was employed to evaluate the overall quality of the literature (Fig. 2), revealing a generally low risk, with the primary concern being incomplete outcome data. For instance, the study by Laidlaw et al.^20^ only included SNOT-22 as an outcome measure, while studies by Bachert^17^ and Fujieda^18^ exhibited issues such as a high dropout rate in the placebo group. Due to the limited number of included studies in this research, a funnel plot was not generated for publication bias detection. Consistency tests indicated that the outcome measures of the studies exhibited no significant inconsistency in both direct and indirect comparisons.

### Network relationship diagram

**Fig. 3:**
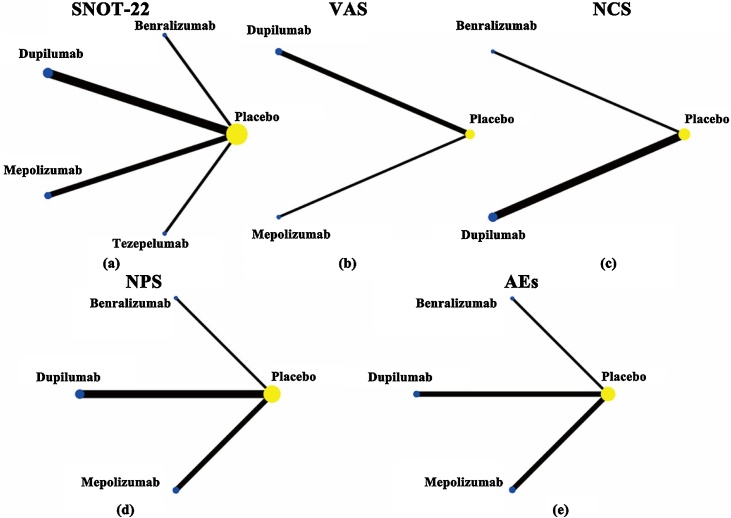
Evidence network relationship diagram: SNOT-22 (a); VAS (b); NCS (c); NPS (d); AEs (e); The thickness of the lines corresponds directly to the quantity of RCTs that evaluate each treatment pair, while the dimensions of each node are in direct proportion to the total number of individuals involved. SNOT-22, Sino-Nasal Outcome Test-22; VAS, visual analogue scale; NCS, nasal congestion Score; NPS, nasal polyp score; AEs, Adverse events.

The network relationship diagram visually represents the interconnections between different interventions. In the diagram, the size of each point corresponds to the sample size of the intervention, while the thickness of the lines is determined by the number of Randomized Controlled Trials (RCTs) associated with each intervention. The comparison results between different biological agents are all derived from indirect comparisons, and the diagram does not contain any closed loops (Fig. 3).

### Results of primary/secondary outcome

**Fig. 4:**
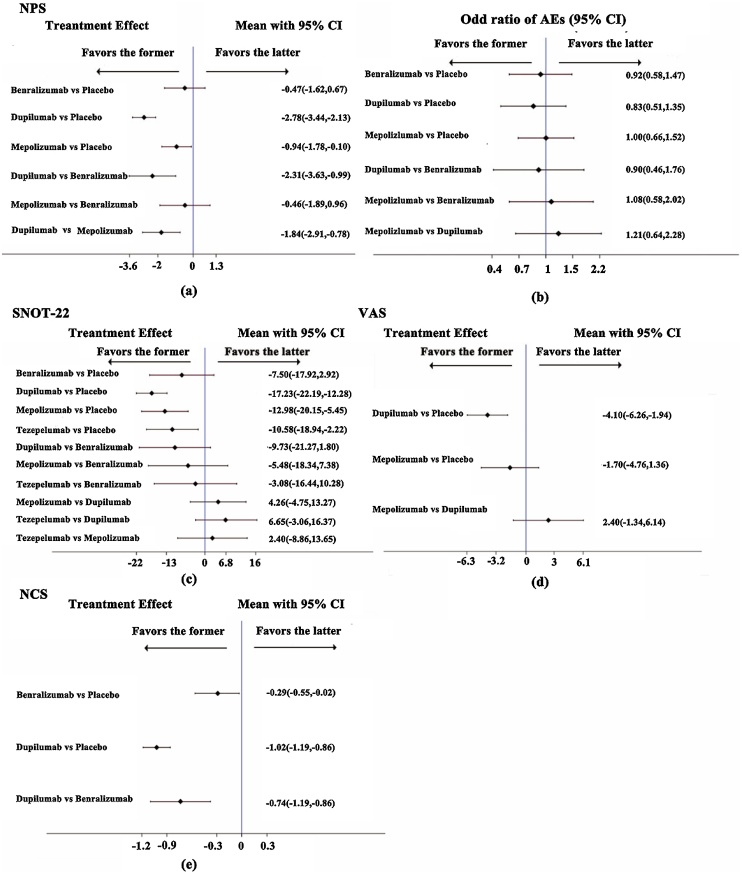
Indirect comparison of primary/secondary outcome measures. (a) Mean difference in NPS score change from baseline to 52-weeks; (b) Different Odd Ratio of AEs of biological agents at 52-weeks; (c‒e) Mean difference in SNOT-22, VAS, and NCS score change from baseline to 52-weeks.

In the long-term treatment of CRSwNP, dupilumab demonstrated superior efficacy in improving NPS compared to other biological agents. At 52-weeks, compared to mepolizumab, benralizumab, and placebo, dupilumab markedly increased the NPS score by 1.84 (95% Confidence Interval [95% CI 0.78, 2.91]), 2.31 (95% CI 0.99, 3.63), 2.78 (95% CI 2.13, 3.44), respectively. Mepolizumab showed a modest improvement in NPS compared to placebo, with an increase of 0.94 (95% CI 0.10, 1.78) (Fig. 4a).

In comparison to placebo, at 52 weeks, dupilumab significantly improved the SNOT-22 scores, VAS scores, and NCS scores by 17.23 (95% CI 12.28, 22.19), 4.10 (95% CI 1.94, 6.26), and 1.02 (95% CI 0.86, 1.19), respectively. Mepolizumab increased SNOT-22 scores by 12.98 (95% CI 5.45, 20.15), while benralizumab increased the NCS scores by 0.29 (95% CI 0.02, 0.55), and tezepelumab improved the SNOT-22 scores by 10.58 (95% CI 2.22, 18.94). Among the comparisons between different biological agents, only dupilumab showed a statistically great improvement in NCS scores compared to benralizumab, with an increase of 0.74 (95% CI 0.86, 1.19), while the comparisons between other biological agents were not statistically significant (Fig. 4c‒e).

Regarding the safety of biological agents at 52 weeks, there were no statistically significant differences in the incidence of long-term adverse reactions between different biological agents and placebo, or between different biological agents (Fig. 4b).

### Ranking of efficacy for different biological agents

**Fig. 5:**
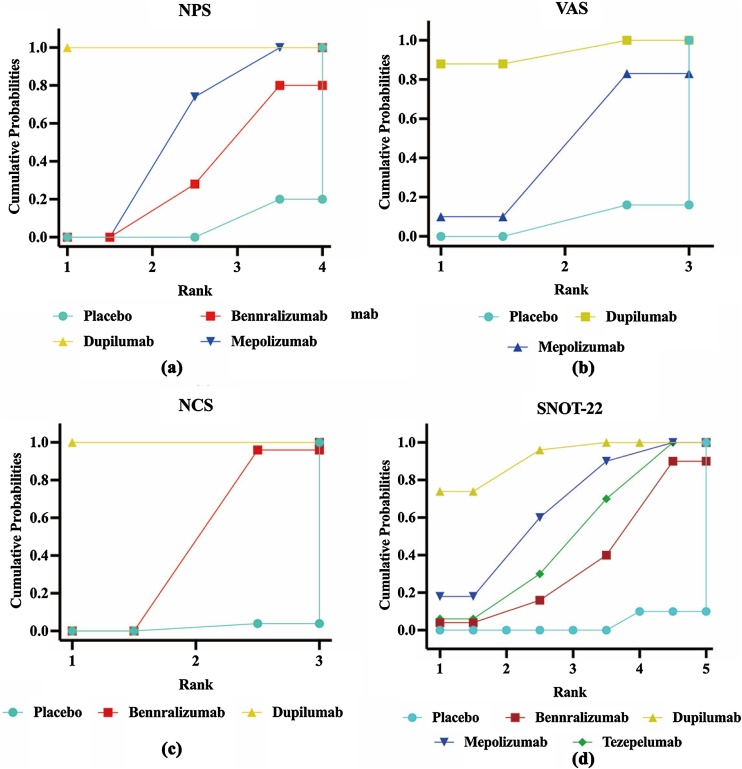
Surface under the cumulative ranking curve for the outcomes: NPS (a), VAS (b), NCS (c), and SNOT-22 (d). NPS, nasal polyp score; VAS, visual analogue scale; NCS, Nasal Congestion Score; SNOT-22, Sino-Nasal Outcome Test-22.

The SUCRA curve was plotted to rank the long-term efficacy of each biological agent (Fig. 5). In terms of NPS, the results indicated that dupilumab had the most favorable efficacy (SUCRA = 100%), followed by mepolizumab (SUCRA = 57.4%). For SNOT-22 scores, dupilumab showed the best efficacy (SUCRA = 72.7%), with mepolizumab following (SUCRA = 42.2%). In VAS scores, dupilumab (SUCRA = 94.6%) exhibited superior efficacy compared to mepolizumab (SUCRA = 48.3%). With respect to NCS, dupilumab (SUCRA = 100%) displayed superior efficacy over benralizumab (SUCRA = 48.0%).

## Discussion

The current network meta-analysis was designed to address the pressing need for comparative long-term efficacy and safety data among various biological agents for the treatment of CRSwNP. In alignment with the European Academy of Allergy and Clinical Immunology’s recommendations,^21^ our study evaluated the long-term outcomes of biological treatments, encompassing six RCTs and a substantial patient population of 1566 individuals. Despite the relatively small number of studies, the literature exhibits a relatively high quality, and the patient population is substantial. Our findings corroborate previous research, with dupilumab emerging as the most effective long-term treatment for CRSwNP.

Our study aligns with previous network meta-analyses,^10^, ^11^, ^12^ which indirectly compared the efficacy and safety of various biological agents for treating CRSwNP over a 24 week period through the analysis of multiple multicenter RCTs. Consistently, dupilumab has been demonstrated to have the most favorable efficacy and safety profile. This finding is reinforced by our analysis, indicating that among CRSwNP cases predominantly characterized by type 2 inflammation, monoclonal antibodies targeting IL-4Rα, which block the IL-4/IL-13 signaling pathway, are the most effective. The positioning of IL-4/IL-13 upstream in the inflammatory signaling pathway suggests that targeting these upstream elements is more efficacious than targeting downstream components, highlighting the critical role of IL-4/IL-13 in the pathophysiology of CRSwNP.^13^

Our study indicates that dupilumab is the most effective long-term treatment for individuals with CRSwNP. Nonetheless, the heterogeneity in inclusion criteria across various studies may lead to variations in the severity and characteristics of inflammation. For instance, studies by Han JK^16^ and Desrosiers^19^ on mepolizumab treatment permitted the use of oral corticosteroids as needed, which could have introduced a bias. The potential discrepancy in corticosteroid use between the monoclonal antibody and placebo groups might have underestimated the true efficacy of mepolizumab. Additionally, the criteria regarding the history of surgical interventions vary across RCTs. The mepolizumab RCT required patients to have undergone at least one sinus surgery in the past decade, suggesting a population with more severe disease given the postoperative recurrences.^22^ In contrast, other RCTs did not specify the extent of previous sinus surgeries. This lack of standardization makes it challenging to compare the efficacy of biological agents across patients who have undergone routine surgeries, such as mini-FESS or polypectomy, with those who have had more invasive procedures like Draf IIb or Draf III, potentially introducing bias into the comparative results.^23^, ^24^

Previous studies have demonstrated differences in the efficacy of different biological agents, with 25%–50% of CRSwNP patients showing no significant benefit from monoclonal antibody treatment, highlighting the importance of selecting patients for biological agents.^24^ However, it remains unclear whether different baseline clinical characteristics at the time of patient enrollment impact the efficacy. Some studies believe that factors such as previous surgical history, asthma status, peripheral blood eosinophil count, IgE, and IL-5 levels do not significantly influence the efficacy of monoclonal antibodies.^4^, ^25^ Conversely, other research found that patients with increased baseline peripheral blood eosinophil levels exhibit better responses to benralizumab and mepolizumab treatment, and that baseline serum osteoprotegerin levels can serve as a biological marker for predicting the response to dupilumab.^26^ In previously published RCTs, only the POLY1 and POLY2 (for omalizumab) selected CRSwNP patients with high IgE levels,^27^ while the remaining trials did not establish corresponding inclusion criteria for type 2 inflammation markers. Therefore, targeting the best biological agent selection based on the patient's immune or clinical characteristics can improve clinical efficacy, future research and guidelines need to address these issues.^28^

Consistent with previous studies, our research found no significant difference in the adverse reactions rate between monoclonal antibodies treatments and placebo. While patients receiving mepolizumab and omalizumab showed a higher incidence of common colds, although it did not reach a significant difference (*p* = 0.05). However, further research is needed to confirm whether biological agents affect the body's immune response to bacteria and viruses.^29^ Additionally, a report based on the global drug safety database indicated a significant association between omalizumab and an increased risk of malignant tumors such as breast cancer and lung cancer.^30^ Hasan et al.^31^ found a significant increase in the risk of skin T-cell lymphoma in patients with atopic dermatitis treated with dupilumab for over one year. Therefore, long-term treatment with biological agents for treating CRSwNP patients should be carefully considered until further research confirms their safety.

### Limitation

Despite the fact that we incorporated six studies that qualified for our criteria, the current analysis is subject to several constraints. Initially, the limited quantity of studies and the modest size of the samples could compromise the robustness and external validity of the findings. Additionally, variations existed across the studies with respect to the initial patient characteristics, therapeutic interventions, and the length of follow-up, potentially introducing bias into the reported outcomes. Furthermore, the majority of the studies originated from economically advantaged countries, and the results may not be generalizable to diverse ethnic and regional patient cohorts. Finally, this analysis did not provide information on personalized treatment strategies based on patients' immunological or clinical profiles, a topic that warrants further investigation. These restrictions are important to consider for a nuanced understanding and practical application of the study's conclusions.

## Conclusion

In summary, our study indicated that benralizumab, dupilumab, mepolizumab, and tezepelumab can all improve important outcome in patients with CRSwNP to varying degrees. Dupilumab appeared to offer a greater advantage in long-term efficacy by reducing NPS and symptom scores compared to other biological agents. Considering the different endotypes among CRSwNP patients from different countries and ethnicities,^32^ future research is needed to identify the optimal biological agents based on patients’ immune or clinical characteristics through large-scale, long-term studies to improve clinical efficacy.

## Registration number/date

This study has been registered on the International Prospective Register of Systematic Reviews (PROSPERO, nº CRD42024540827).

## CRediT authorship contribution statement

Han Chen: Conceptualization; methodology; validation; formal analysis; writing-original draft.

Lin Wang: Software; Validation; formal analysis; investigation; writing-review & editing.

Jisheng Zhang: Software; validation; formal analysis; investigation; writing-review & editing.

Xudong Yan: Investigation; data curation; writing-review & editing.

Longgang Yu: Conceptualization; methodology; writing-review & editing; supervision.

Yan Jiang: Conceptualization; methodology; writing-review & editing; supervision; funding acquisition.

## Ethics approval and consent to participate

An ethics statement is not applicable because this study is based exclusively on published literature.

## Funding

This work was supported by grants from the Program for Natural Science Foundation of Shandong Province (ZR2023MH027), Medicine and Health Science Technology Development Program of Shandong Province (202207010780), and Natural Science Foundation of Qingdao Municipality (23-2-1-199-zyyd-jch).

## Data availability statement

The data used to support the findings of this study are included within the article.

## Declaration of competing interest

The authors declare no conflicts of interest.
